# The Reason Why the Dental Department of the University of Maryland Deems It Inexpedient to Join the National Association of Dental Faculties

**Published:** 1885-07

**Authors:** F. J. S. Gorgas

**Affiliations:** Dean of University of Maryland, Dental Department.


### EDITORIAL, ETC.

---

THE REASON WHY THE DENTAL DEPARTMENT OF THE UNIVERSITY OF MARYLAND DEEMS IT INEXPEDIENT TO JOIN THE NATIONAL ASSOCIATION OF DENTAL FACULTIES, IS SET FORTH IN THE FOLLOWING LETTER:

*University of Maryland, Faculty of Physic, Department of Dental Surgery.*

BALTIMORE, July 28th, 1885.

*To the National Association of Dental Faculties.*

GENTLEMEN:

The faculty of the University of Maryland, Dental Department, desire to acknowledge the receipt of the "Transactions of the National Association of Dental Faculties," and also the notice of the meeting to be held in Chicago, July 31, 1885, with Article VII. of the constitution.

I am directed by the Faculty of the University of Maryland, Dental Department, to inform your Association, that they will careerfully comply with all the requirements relating to the graduation of students in dentistry, as adopted at the meetings held in New York City and Saratoga in August, 1884, so far as refer to two full courses of five months each in sepaiate years, etc., and have published the same in our Annual Catalogue of 1885, copies of which have been mailed to the President and Secretary of your Association. At the same time, however, the Faculty of the University of Maryland, Dental Department, deem it inexpedient to join the National Association of Dental Faculties, for the reason that they believe the present curriculum of study, etc, in the University of Maryland, to be superior to any graded course of study, such as is obligatory upon all dental schools joining your Association, for the reason that the graded course restricts junior students to dental mechanism alone for the one session of the two comprising the full course to the exclusion of operative dentistry, and, therefore, affords dental students the advantage of but one session in the acquirement of a knowledge of a branch of our science for which the time of two entire sessions is not too long.

The Faculty of the University of Maryland, Dental Department, contend that students in dental schools should have all the advantages possible of the two sessions in operative as well as in mechanical dentistry, and that the adoption of such a graded course as that required by your Association, and which restricts the dental student in the manner referred to, must be *retrogressive* instead of progressive in dental education. Respectfully, &c.

F. J. S. GORGAS.

Dean of University of Maryland, Dental Department.

